# U.S. Cooperative Extension's response to substance misuse: A scoping review

**DOI:** 10.3389/fpubh.2023.1127813

**Published:** 2023-02-17

**Authors:** Angela Hagaman, Kristen Roark, Lisa Tucker Washburn

**Affiliations:** ^1^Addiction Science Center, College of Public Health, East Tennessee State University, Johnson City, TN, United States; ^2^College of Public Health, East Tennessee State University, Johnson City, TN, United States; ^3^Institute of Agriculture, University of Tennessee, Knoxville, Knoxville, TN, United States

**Keywords:** substance abuse, cooperative extension, drug abuse, opioid, prevention

## Abstract

**Background:**

The U.S. has experienced exponential growth in overdose fatalities over the past four decades and more than 22 million people are currently living with a substance use disorder (SUD). While great strides have been made in advancing the science of SUD prevention and treatment, proven programs and interventions are not commonly disseminated at scale in impacted communities. The U.S. Cooperative Extension System (Extension) has been recognized as a valued partner in addressing SUD in communities. Federal funding supporting Extension's response to the opioid epidemic reached $35 million in 2021 primarily through two grant programs: the United States Department of Agriculture's (USDA) Rural Health and Safety Education program; and the Substance Abuse and Mental Health Services Administration (SAMHSA) Rural Opioid Technical Assistance (ROTA) grants. The primary objective of this scoping review was to identify the range of Extension activities aimed at mediating substance misuse.

**Methods:**

Authors utilized the PRISMA-SCR model to complete this scoping review. Due to the nature of Extension work and the expectation that few activities would be cited in the peer-reviewed literature, the scoping review included a search of peer-reviewed databases, Extension websites for each state and U.S. territory, and the utilization of a web search engine. Upon initial analysis of records returned, authors noted a discrepancy between results returned and the number of states receiving ROTA grants. Thus, authors supplemented the PRISMA-SCR review protocol with a systematic procedure for investigating ROTA funded activities not readily apparent in the peer-reviewed or grey literature.

**Results:**

A total of 87 records met inclusion criteria. Findings included seven peer-reviewed articles and 80 results from the grey literature. An additional 11 ROTA grantees responded to requests for information regarding state level activities.

**Conclusions:**

Nationwide, Extension has scaled multiple efforts to address SUD operating through a loose confederation of organizations connected to the land-grant system. Most activities are funded by federal grants and focus on state-sponsored training and resource sharing. The volume of effort is significant, however, implementation at the community-level has been slow. Significant opportunities exist for local adoption of evidence-based practices aimed at mitigating SUD.

## Introduction

Overdose fatalities in the U.S. have continued to rise over the past four decades (1). Substance use and misuse are contributing to an escalating burden of disease leading to premature death and disability (2) and a host of other associated harms including childhood and family trauma (3), excessive health care costs (4), and justice system involvement (5). An estimated 174 people die each day in the U.S. from a drug-related overdose (1) and more than 22 million people are currently living with a substance use disorder (SUD) (6). In April of 2021, in the midst of the COVID-19 pandemic, the U.S. surpassed 100,000 drug-related deaths in a 12-month period for the first time in history (7).

Concurrent to these year-on-year increases in overdose fatality, numerous advances have been made in the science of SUD prevention and treatment. Unfortunately, many of these proven programs and interventions are not disseminated to scale in impacted communities to counter the mounting burden of disease. Cited barriers to the dissemination of evidence-based programs and policies include poor translation and clarity of scientific studies (8), perceived patient factors, limited community-level referral sources (9), stigma, and other systemic barriers (10). Moreover, communities most affected by SUD, such as those in rural areas or the Appalachian region, may also be communities with limited resources, and thus, the effective dissemination and implementation of preventative interventions may prove more challenging.

The Cooperative Extension System (Extension) is part of the land grant university system in the United States. Extension is a nationwide network of local offices affiliated with and partially supported by land grant institutions with offices in or near most U.S. counties (11). Local Extension offices house teams of educators who offer educational outreach and programs in several areas, including agriculture and natural resources, 4-H and youth development, family and consumer sciences, and community economic development. Extension efforts are informed by local priorities and needs and shaped by programmatic resources available from the sponsoring land grant institution. Extension personnel serve as vital partners across multiple community-based efforts and more recently have been recognized as valued partners in addressing SUD and related issues impacting families and communities (12). To that end, the Extension Opioid Crisis Response Workgroup was formed in February 2018 at the recommendation of the Extension Committee on Organization and Policy and following the declaration of the opioid crisis as a national public health emergency in 2017 (13). The purpose of this group was to identify and organize resources to help Extension play a clear and intentional role in addressing the opioid crisis and general behavioral health challenges (14). Moreover, a 2018 survey of national Extension leaders indicated that an overwhelming majority agreed that Extension should play a role in reducing opioid misuse and overdose in their respective states, but <24% agreed their Extension System had capacity to respond to the epidemic (14).

Federal funding supporting Extension's response to the opioid epidemic exceeded $35 million between 2017 and 2021 (15, 16). This funding originated primarily through the United States Department of Agriculture's (USDA) Rural Health and Safety Education program and the Substance Abuse and Mental Health Services Administration (SAMHSA) Rural Opioid Technical Assistance (ROTA) grants. Additional funding was also provided through USDA's Children, Youth and Families at Risk grant program (17). For nearly 6 years, Federal funding has prioritized Extension projects and trainings that address the opioid crisis, however, the full scope of this work and related outcomes is unknown.

The primary objective of this scoping review was to identify the full range of Extension activities aimed at mediating opioid and substance misuse from 2016–2022. Furthermore, the review may help to elucidate opportunities for the expansion of existing programs and assets or fill gaps and implement programs in areas that are not currently working in this space, specifically, those in rural communities and those most impacted by SUD.

## Materials and methods

### Study design and search strategy

This scoping review was conducted from February through July of 2022 and was informed by the PRISMA-SCR (Preferred Reporting Items for Systematic Reviews and Meta-Analyses extension for Scoping Reviews) model (18). Due to the nature of Extension work, authors anticipated that few activities would be cited in the traditional peer-reviewed literature, thus, the review included searches within peer-reviewed databases and within one standard web search engine. First, authors used combinations of the following search terms to query the peer-reviewed literature *via* PubMed, EBSCOhost, ProQuest, Elsevier, and JSTOR: opioid, cooperative extension, drug, substance, and substance abuse. Authors also utilized the OneSearch platform hosted by the East Tennessee State University's Sherrod Library to expand the search to additional databases. Additional filters applied during the search included: (1) peer-reviewed, (2) full article, and (3) 2016 and newer.

Following a search of the peer-reviewed literature, authors queried Extension websites for each state and U.S. territory and utilized the Google search engine using combinations of the following key terms: opioid, cooperative extension, drug, substance, and substance abuse. Additional filters included records that were written in English and published between 2016 and 2022. Upon conclusion of the initial search and saturation of the traditional literature and web findings, a preliminary review of records excluded duplicates and records or titles that were not connected to Cooperative Extension and/or were not focused on SUD or opioid related topics. In this preliminary review, records were included only if they met all of the following criteria: (1) connection to Extension work; (2) focus on programs or activities designed to mediate opioid or substance misuse; (3) written in English; (4) and published between the years of 2016 and 2022. Each included record was then documented in an Excel spreadsheet and sorted into two categories: peer-reviewed and web results. Authors then classified the web results as press releases, news articles, Extension blogs or feeds, impact statements, and online videos or online training series, and defined these records for the purposes of this review as “grey literature” (19).

**Figure 1:**
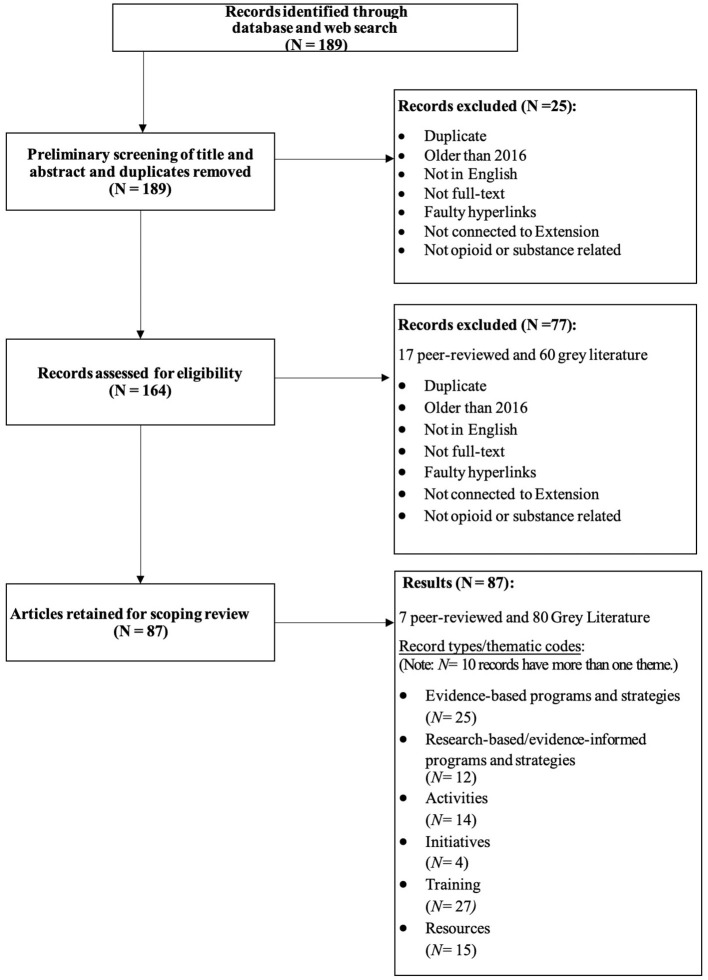
Scoping review flow chart.

A pair of reviewers then conducted a critical analysis of each record to ensure that each still met the inclusion criteria. Reviewers also began coding records into themes or result “types.” The reviewing team met on three separate occasions to discuss discrepancies in the thematic codes and any newly excluded results. The Excel spreadsheet was updated following each meeting. Upon the third review, a final set of six themes was established for the grey literature and included: evidence-based programs and strategies, research-based and evidence-informed programs and strategies, activities, initiatives, training, and resources. Figure 1 illustrates the comprehensive review process.

### Supplemental analysis

Upon conclusion of the records review process, the authors identified a notable discrepancy in the number of records returned as compared to the number of states that received opioid focused grant funds. Twenty-three states received ROTA funding during the years 2016-2022, however, only 9 records identified this funding explicitly *via* the PRIMSA-SCR informed scoping review process. Thus, the authors agreed to supplement the PRISMA-SCR protocol with a systematic *post-hoc* procedure for investigating ROTA funded activities not readily apparent in the peer-reviewed or grey literature and to identify the full range of Extension activities aimed at mediating opioid and/or substance misuse. The authors developed a standard email inquiry and interview guide and then contacted all ROTA-funded state project directors with a request for a brief telephone interview to confirm project activities and scope utilizing a pre-defined set of questions.

## Results

The initial search of the peer-reviewed and grey literature resulted in 189 returned records. Following a preliminary review to identify duplicates and titles that did not meet inclusion criteria, 25 records were excluded, and 164 records were retained (22 peer-reviewed and 142 web/grey literature). The next stage in the process included a critical appraisal of each remaining record by two of this paper's authors. This phase included three iterations of review and thematic coding of each record by type. An additional 77 records that did not meet inclusion criteria were removed in this phase of the review. Upon completion of the full review process, final results included 87 records (80 web/grey literature and 7 peer-reviewed). Moreover, ten (9) records found in the grey literature search overlapped across multiple themes.

### Peer-reviewed results

The seven peer-reviewed articles included were thematically categorized as: (1) Conceptual-System - focused on the Cooperative Extension System, capacity, and potential as it applies to SUD prevention, education, or behavioral health; (2) Interventions - described evidence-based interventions, approaches, or results; and (3) Coalitions- focused on coalition-based approaches and support; (4) Needs Assessment; and (5) Supplemental.

Two of the seven peer-reviewed results were coded as Conceptual-System. The first provided a conceptual framework for Extension's response to the opioid crisis with examples of Extension professionals as public health agents bringing practical, evidence-based solutions to communities to combat increases in poly-substance use (20). The second provided a framework for science-driven behavioral health translation to more effectively address substance misuse through the Extension system (21).

Two results were coded as Interventions. One record focused on the evidence-based PROSPER system (PROmoting School-Community-University Partnerships to Enhance Resilience created for implementation through Cooperative Extension (22). A second article described a multi-state education and outreach effort including opioid misuse prevention programming in rural communities (23).

A fifth record from the traditional literature focused on coalition-based approaches as a promising approach for addressing disparities in rural health such as substance misuse (24). The sixth record was categorized as a Needs Assessment and described community-focused needs assessment conducted as part of the PROMISE (Preventing Opioid Misuse in the SouthEast) initiative (25). The final peer-reviewed article was deemed Supplemental and included in this review due to the broad focus on 4-H Healthy Living programs, which are widely implemented through Extension across the U.S. This article described an evaluation study reviewing 32 promising health-related positive youth development programs, some of which included prevention of alcohol, tobacco and other drug use (26).

### Grey literature results

A total of 80 records were included in the grey literature results. Sources meeting inclusion criteria were representative of Extension in nearly every state except Arizona, Connecticut, Maine, New Jersey, and South Carolina. Authors categorized the grey literature into six coded themes: Evidence-based Programs and Strategies, Research-based and Evidence-Informed Programs, and Strategies. Activities, Initiatives, Training, and Resources. Most states' opioid response activities included a combination of efforts across categories. The paragraphs below summarize findings by thematic code. Table 1 contains a more detailed description of each result including states implementing and all related citations.

**Table 1:** A description of findings from the grey literature review.

**Theme/Type**	**Title**	**Description**	**Implementing states**
Evidence-based programs and strategies	Mental health first aid (*N=* 9)	Skills-based training course that teaches participants to identify, understand, and respond to mental health and substance use challenges.	Indiana (27) Ohio (28, 29) Maryland (30) Mississippi (31) Oregon (32) Texas (33) Virginia (34) Wisconsin (35)
Evidence-based programs and strategies	Chronic pain self-management program/chronic pain PATH (Personal action toward health) (*N=* 3)	6-session small group workshop teaching techniques to deal with symptoms of chronic pain with emphasis on cognitive behavioral therapy techniques.	Arkansas (36) Michigan (37) New Hampshire (38)
Evidence-based programs and strategies	PROSPER Delivery System (Promoting School-community-university Partnerships to Enhance Resilience) *(N=* 3)	Delivery system linking Extension with public schools; facilitates delivery of evidence-based programs that reduce risky youth behaviors.	Ohio (29) Virginia (39) Vermont (40)
Evidence-based programs and strategies	Botvin's Life Skills Training (LST) (*N=* 3)	Classroom-based youth program promoting healthy alternatives to risky behavior.	Kentucky (41) Virginia (39) Vermont (40)
Evidence-based programs and strategies	Strengthening Families 10–14 (*N=* 4)	Program for youth ages 10–14 and their parents; taught with 7–10 families over seven weeks of in-person sessions.	Iowa (42) Virginia (39) Vermont (40) Wisconsin (35)
Evidence-based programs and strategies	Raising a thinking child (*N=* 1)	8-week parenting program for parents and caregivers of children 4–7 years; developing interpersonal cognitive problem-solving skills, improving parenting skills and parent-child communication, and in decreasing impulsive and inhibited behaviors in young children.	Wisconsin (35)
Evidence-based programs and strategies	Communities that care (*N=* 1)	Community-driven prevention process promoting healthy youth behavior and universal prevention approaches.	Tennessee (43)
Evidence-based programs and strategies	WISE: Wellness initiative for senior education (*N=* 1)	Educates older adults on variety of topics including safe medication use and avoiding misuse.	Michigan (44)
Research-based /evidence-informed programs and strategies	Empowering youth and families (*N=* 1)	10-week program focused on opioid prevention education for youth and their caregivers.	North Carolina (45)
Research-based /Evidence-informed programs and strategies	Generation Rx (*N=* 1)	Educational initiative providing resources and training on safe medication use.	Ohio (46)
Research-based /Evidence-informed programs and strategies	4-H health rocks! (*N=* 1)	Program teaches life skill development and decision-making to reduce tobacco, alcohol, e-cigarette/vaping and drug use. Uses Teens-as-Teachers model.	Illinois (47)
Research-based /Evidence-informed programs and strategies	Recovering Your Finances (*N=* 1)	8-week program specifically addresses financial issues those in recovery may face and guidance for overcoming those obstacles.	Kentucky (41)
Research-based /Evidence-informed programs and strategies	Youth Advocates for Health (*N=* 1)	Take-PART (Participatory Action Research with Teens) Opioid Research Project Internship in partnership with Upward Bound.	Washington (48)
Research-based /Evidence-informed programs and strategies	Good drugs gone bad (*N=* 1)	Program encouraging safe disposal and safe storage of prescription medications.	Wisconsin (35)
Research-based /Evidence-informed programs and strategies	4-H: Your life, your health (*N=* 1)	5-session educational series addressing underage drinking, drinking and driving, illegal substance abuse and communication skills in families.	North Carolina (49)
Research-based /Evidence-informed programs and strategies	CADA Youth Training - Agents 4 Change (*N=* 1)	Youth-focused training helps youth be community change agents by understanding community engagement and how to organize community change.	Wisconsin (50)
Research-based /Evidence-informed programs and strategies	Mind.Art.Recovery. KY (*N=* 1)	Expressive arts-in-health curriculum piloted with those in rehabilitation and at-risk facilities.	Kentucky (41)
Research-based /Evidence-informed programs and strategies	Community first responder program (*N=* 1)	Teaches members of the community to recognize the signs of overdose and how to respond.	Rhode Island (51)
Research-based /Evidence-informed programs and strategies	Stress less with mindfulness (*N=* 1)	5-session program teaching the experience and practice of mindfulness to reduce stress.	Michigan (52)
Research-based /Evidence-informed programs and strategies	Move with Ease (*N=* 1)	6-week program that teaches chronic pain participants to practice poses that help improve range of motion.	Arkansas (36)
Activities	Project STOMP (*N=* 1)	Youth-focused prevention awareness campaign engaging 6-12th graders to produce PSAs addressing substance misuse.	Iowa (53)
Activities	Community Conversations (*N=* 2)	Public meetings to share information about activities or solicit feedback from community members about local needs related to SUD.	Ohio (46) Oregon (32)
Activities	Stakeholder Engagement in Question Development and Prioritization (*N=* 1)	Multi-stakeholder approach to engaging communities in research, problem solving, and action planning.	Virginia (39)
Activities	Opioid education dinners (*N=* 1)	Dinner and discussion about the current state of the opioid crisis and real-world ways to support loved ones and get the help you need.	Utah (54)
Activities	Drug take-backs, drug drop boxes (*N=* 2)	Prescription drug drop off programs and sharps disposal options.	Mississippi (55) Wisconsin (35)
Activities	Coalitions engagement and support (*N=* 3)	Partnerships with community coalitions and engagement and support from academic departments.	Kentucky (41) Ohio (46) Wisconsin (35)
Activities	Fentanyl test strip distribution (*N=* 1)	Partnership with the College of Pharmacy to distribute fentanyl test strips to both clinics and community-based organizations that focus on harm reduction.	Minnesota (56)
Activities	Naloxone kit distribution(*N=* 2)	Distribution of naloxone opioid overdose reversal kits in multiple venues.	Minnesota (56) Rhode Island (51)
Initiatives	Recovery friendly workplaces (*N=* 1)	Webpage shares the results of a statewide survey of MO business owners and also links to the Missouri Recovery Friendly Workplace program.	Missouri (57)
Initiatives	ADA training for employers (*N=* 1)	Poster presentation at the Ohio State University Community Engagement Conference entitled *Employment and the Opioid Crisis in Ohio: How Extension, Community Partners and the American with Disabilities Act Can Support Local Employers*.	Ohio (58)
Initiatives	Healthy grandfamilies (*N=* 1)	Healthy Grandfamilies is a free initiative led by West Virginia State University to provide information and resources to grandparents who are raising one or more grandchildren.	West Virginia (59)
Initiatives	Nutrition education and gardening projects (*N=* 1)	Nutrition education and gardening project implemented in partnership with local recovery center.	West Virginia (60)
Training	Recorded trainings (*N=* 4)	Webinars or recordings of virtual events.	Maryland (61) Montana (62) Michigan (52) Rhode Island (63)
Training	Naloxone/Narcan (*N=* 3)	Training on administering naloxone or Narcan delivered to community members, law enforcement, and other groups.	Rhode Island (63) South Dakota (64) Wisconsin (65)
Training	Drug disposal and safe storage education (*N=* 3)	Educational sessions sharing information on safe storage of drugs and disposal methods.	Mississippi (66) Montana (67) Oklahoma (68)
Training	Opioid and SUD awareness, stigma reduction (*N=* 8)	Training delivered through a variety of formats (live webinars, in person programs, recorded webinars, and voice over PowerPoint) provided to increase awareness of the opioid crisis and SUD, and decrease stigma. Audiences included Extension professionals, healthcare providers, and parents, and community members.	Indiana (27) Kentucky (41) Montana (62) Minnesota (69) Oklahoma (68) Oregon (32) South Dakota (64) Tennessee (43)
Training	Continuing education for healthcare providers (asynchronous or live virtual Project ECHO) (*N=* 5)	Variety of training topics many with CE credit on topics such as harm reduction, treatment referral, non-pharmalogical approaches to pain management, and stigma.	Kentucky (41) Michigan (44) New Hampshire (38) Utah (70) Washington (71)
Training	De-stigmatizing Media training (*N=* 1)	Provides best practices for covering substance use disorder prevention, treatment, and recovery through 90-minute virtual sessions.	Oregon (32)
Training	Patient education - opioid risks (*N=* 1)	Target hospital patients to help make them aware of the dangers associated with opioid pain medications.	Virginia (39)
Training	Trauma-Informed Care Training (*N=* 2)	Training to increase understanding of Adverse Childhood Experiences (ACEs) and the impact of trauma.	Ohio (72) Wisconsin (65)
Resources	Story telling (*N=* 5)	Podcasts, audio project, rural radio programs, recovery stories, video PSA	Michigan (52)Montana (62) Oregon (32) Utah (73, 74)
Resources	Your thoughts matter 4-H project book (*N=* 1)	Project book that offers hands-on learning for adolescents and meets National Learning Standards.	Ohio (29)
Resources	Fact sheets (*N=* 6)	-Stigma -Prevention -Understanding addiction and treatment -Adolescent substance use -Opioids in rural farming communities	Colorado (75) Delaware (76) Michigan (52) Minnesota (77) Mississippi (66) Tennessee (78)
Resources	Drug disposal (*N=* 2)		Mississippi (66) Oklahoma (79)
Resources	4-H Opioid display (*N=* 1)		Ohio (46)

### Evidence-based programs and strategies

The PRISMA-SCR informed search located eight evidence-based programs/strategies being implemented through the Extension system to address aspects of opioid misuse. Records for eight states implementing Mental Health First Aid were found. States implementing the PROSPER (PROmoting School-community-university Partnerships to Enhance Resilience) model also implemented two evidence-based programs as part of the PROSPER delivery system, one youth- and one family-focused program. Most often Botvin's Life Skills Training and Strengthening Families 10-14 were paired in PROSPER states. Several states offered chronic pain programs including Chronic Pain PATH and the Chronic Pain Self-Management Program, reaching underserved adults in rural areas.

### Research-based and evidence-informed programs and strategies

The grey literature also returned 14 research-based and evidence-informed programs implemented through Extension. Programs ranged from multi-week, multi-session education programs for youth and families to training community members in opioid overdose response. Content included nutrition education, financial management, and physical activity. Some incorporated community engagement approaches, while others focused primarily on increasing knowledge or awareness.

### Activities

Search results also demonstrated a range of activities that have been implemented through Extension in the U.S., including naloxone kit and fentanyl test strip distribution, awareness-raising community dinners and other community forums, and support for coalition-engaged approaches. Activities are sometimes paired with other educational strategies, like providing training on naloxone use prior to providing naloxone kits. Activities are often conducted with partner organizations (e.g., drug takeback events or drop box placement in collaboration with law enforcement).

### Initiatives

The scoping review process yielded two records specifically focused on the workplace. The Recovery Friendly Workplaces initiative implemented in one state helps employers support people affected by SUD and those in recovery. This initiative focuses on creating a workplace culture that promotes employee safety, and health and wellbeing while reducing stigma and providing recovery resources. Another state's initiative focused on employer training dedicated to the Americans with Disabilities Act, including protections for those in recovery. Moreover, this review found several projects offering existing subject-matter expertise and resources to those in recovery and their families through local organization partnerships. Two states conducted nutrition education and implemented gardening projects in partnership with local recovery centers. Finally, as parenting and family life education are traditional areas of Extension outreach, the Healthy Grandfamilies initiative extends this focus to include the provision of information and resources to grandparents who are raising one or more grandchild due to a family members' challenges with substance use disorder.

### Training

Results in this category indicated that training was provided on a range of topics with varied target audiences using synchronous, asynchronous, and hybrid methods. Training topics included administering naloxone to community members, law enforcement, and other groups. SUD and opioid awareness topics, including stigma reduction, were also delivered through a variety of formats (live webinars, in person programs, recorded webinars). Audiences included Extension professionals, healthcare providers, parents, and community members. Some states provided continuing education for healthcare providers, both asynchronously and through live virtual sessions utilizing Project ECHO. Training topics included trauma-informed care and other more targeted training, such as a De-stigmatizing Media Training that provides best practices for covering SUD prevention, treatment and recovery. Finally, one state's patient education program addresses opioid risk and provides additional education sessions on safe storage and disposal of drugs.

### Resources

A number of states produced a range of educational resources to raise awareness and reduce stigma. Several educational video series offered introductory information about substance use for the public, and other resources included personal stories of people impacted by substance misuse. Recovery resources were also provided including podcasts, audio recordings, videos, and one rural radio program. Web and print publications covered topics including prevention, stigma, understanding addiction and treatment, adolescent substance use, and opioids in farming communities. Some resources supplemented training topics such as prescription drug disposal or resources aimed at youth audiences such as displays and 4-H project books. Social media marketing messages were also developed and included in one statewide campaign.

### Supplementary findings from grantee interviews

As discussed previously, the authors of this paper universally agreed that the nine records returned *via* the PRISMA-SCR search process were insufficient to illustrate the contributions of the 23 states funded by ROTA grants during the six-year time frame for this search. Eleven states responded to the request for interview and the grant program director, or a designee, participated in a 30-min interview by phone or Zoom to answer the pre-defined list of questions. Responses frequently aligned with the six previously identified grey literature themes. The following responses were consistent with the evidence-based programs and strategies theme: Question, Persuade, Refer in Oregon; WeCOPE (Connecting with Our Positive Emotions) in Wisconsin, and CRAFT: Community Reinforcement and Family Training in South Dakota. Virginia also implemented two evidence-based programs: Botvin's Life Skills Training Parent Program and Too Good for Drugs, a universal K-12 prevention education program. Research-based/evidence-informed programs found *via* the supplementary interview process included RELAX: Alternatives to Anger in Michigan, and Learning to Breathe, a mindfulness curriculum for youth, in Wisconsin.

A unique approach implemented in Oklahoma included partnerships with rural fire departments for naloxone education and distribution and aligned with the Initiatives theme. This state relied upon coalition engagement and hired field faculty specifically dedicated to opioid response. A Virginia initiative included hiring regional coordinators to work with coalitions in multiple counties. Several interviewees revealed that their project approaches evolved over time when new data became available thereby informing improvements to their funded strategies. ROTA project interviewees also described newly developed Training tools, such as Montana's Prescription Opioid Toolkit, a 5-module packaged curriculum for Extension field agents use in communities.

## Discussion

This scoping review is the first, to our knowledge, to compile efforts of the U.S. land-grant university Extension system to address the opioid crisis. The results offered in this PRISMA-SCR informed review (18) highlight a ramp-up of Extension activities following the 2016 shift in federal funding priorities. However, continued and expanded engagement of Extension personnel is still warranted given the far-reaching impacts of the opioid epidemic and ongoing challenges of SUD and overdose fatality for families and communities. Expanded engagement may be particularly impactful in rural areas disproportionately affected by SUD and where Extension may serve as an active partner in agricultural activity, and thus, may have increased credibility when engaging in community-based SUD initiatives.

University-generated publicity announcing federal grant awards represented a large proportion of the grey literature sources identifying opioid related Extension activities. This information underscores the critical role of federal funding priorities in driving the initiation or expansion of SUD related prevention and recovery activities in many states. Authors are unable to speculate whether states would have pursued the same volume of activities absent external funding support, and to our knowledge, there no studies investigating this topic. Given Extension's limited capacity and resources to address SUD prior to funding availability, it seems unlikely this range of activities would have occurred. The combination of funded activities involving both mental health and SUD illustrates how specific states repurposed or reframed existing programs and traditional Extension content (e.g., stress coping, mindfulness, parenting) to reach new audiences. This approach was likely influenced by evolving ROTA funding requirements which required integration of mental health and SUD.

Few partnerships between Extension and non-Extension community partners were identified in the initial scoping review process. However, findings gleaned through supplemental interviews with ROTA grantees confirmed collaborative efforts and state/regional agency partnerships in opioid response. For example, a social marketing project developed and tested through an Extension partnership with other university academic units primed a larger campaign disseminated by a state agency. This social marketing content originated through Extension effort but would have been unidentified using PRISMA-SCR methods. Collaboration, while essential to opioid response efforts, may have made identifying Extension contributions more difficult. This, however, is probably a necessary by-product of true collaboration where accomplishing the work is more important than naming the headlining or sponsor agency.

Moreover, interviews with ROTA grantees provided context for opioid-focused activities that could not have been obtained through traditional scoping review methods. For example, partnerships with state agencies and community organizations not apparent through grant award announcements and project websites were vital to understanding overall project implementation. These interviews also provided information on implementation processes and barriers beyond the scope of this review but worthy of further exploration. Many interviewees found it difficult to attribute specific activities to a particular funding source or funding period. A series of prerequisite grant funding determined state's initial eligibility for ROTA funding; thus, interrelated grant funded activities were inherent. Work under these funding sources intersected and became interwoven, suggesting movement toward institutionalizing opioid and SUD related activities as opposed to simply adhering to grant timelines and objectives. Implementation of opioid-focused activities that was not required by grant funding also suggests increased acceptance of Extension's role in opioid misuse prevention and other SUD related topics.

Extension is typically located in Colleges of Agriculture within land grant universities. This traditional organizational structure and related subject-matter expertise of the Extension-affiliated faculty specialists may limit the capacity to support work occurring locally. This structure may also hamper visibility of activities addressing SUD by making efforts harder to recognize within the larger university system. A concentrated effort by Federal funding entities to develop formal dissemination strategies for grant-funded outcomes at the local level could address this challenge. This search found limited but intentional linkages between multiple academic units working to impact SUD. Such approaches bridged pharmacy, social work, nursing, and behavioral science disciplines to enhance activities beyond Extension's traditional subject matter focus and thereby potentially expanding overall impact at the organizational and systems levels. Finally, partnerships with external entities, such as state agencies and state or local non-profit organizations, appeared to broaden the reach of Extension activities, thereby strengthening the collective response of the Extension System to the opioid crisis.

## Limitations

The PRIMSA-SCR method created a foundation from which to initiate the scoping review, however, it was insufficient to achieve the author's goals, thus requiring the adoption of supplemental methodology (e.g., state-level interviews) to identify the range of Extension activities aimed at mediating substance misuse. Moreover, the inclusion criteria for this search, while appropriate given the focus of this scoping review, may have excluded relevant results broadly related to SUD. Initial shifts in federal funding priorities for Extension specifically related to the opioid crisis, thus, authors may have prioritized data sources specific to opioid misuse. Records returned limited grey literature results beyond those described in the initial funding announcements. However, direct contact with ROTA grantees revealed a range of activities and resources. Thus, a key limitation in our search may be characteristic of the organization of focus (Extension) whereby associated strategies for promotion and marketing may result in infrequent website updates and lack of keywords for search engine optimization. Further limitations are also inherent within the U.S. Extension System. For example, publication in peer-reviewed journals, while standard for disseminating knowledge in academia, is less common among Extension professionals, particularly those focused on local implementation. Furthermore, time parameters for the search may have limited search returns and these results can only be considered current through July 2022. Time needed to produce results, COVID-19 implementation delays, and long wait times for peer-reviewed publication may also explain why few traditional journal articles were found.

Finally, the search methods utilized in this review may have unintentionally excluded efforts of historically black and Native American tribally controlled land-grant institutions. Federal legislation established or extended land-grant status to these institutions in 1890 and 1994, respectively, and they share the Extension mission (80). While selected cooperative activities between land-grant universities were found, particularly in states with tribal colleges, we acknowledge that results may not be fully representative of all work occurring, particularly through institutions with university names not including, “cooperative extension,” or other specified search terms.

## Conclusions

This is, to our knowledge, the first review of its kind to provide a snapshot of Extension's efforts to address the opioid and SUD crisis in the U.S. While these results may not include every Extension activity that occurred during the specified time frame, Extension has significantly increased efforts to address the opioid crisis and related challenges operating through a loose confederation of organizations that are part of the land-grant system. Numerous training activities, resources, and toolkits have been developed at the state level through funding by federal grant dollars. The volume of effort is significant. Continued efforts and expanded partnerships, particularly for community-level work, are needed to combat the ongoing epidemic of opioid and substance misuse across the country.

## Data availability statement

The original contributions presented in the study are included in the article/supplementary material, further inquiries can be directed to the corresponding author.

## Author contributions

AH contributed to the conception and design of the study, reviewed and coded all search results, assisted with writing the introduction, constructed Table 1 and edited Figure 1, inserted all citations, and was responsible for combining, and proofreading all sections of the manuscript. KR conducted the initial web and peer-reviewed literature search, built the excel file for co-authors to review, prepared Figure 1, and assisted with writing results. LW was the Principal Investigator of the Rural Health and Safety Education project (grant no. 2019-46100-30276) from the USDA that funded this work, contributed to the conception and design of the study, reviewed and coded all search results, assisted with writing the introduction, assisted with compiling the results, drafted the discussion, and conclusion sections. All authors contributed to manuscript revision, read, and approved the submitted version.
